# Heads-up 3D vision system for retinal detachment surgery

**DOI:** 10.1186/s40942-017-0099-2

**Published:** 2017-11-20

**Authors:** Michele Coppola, Carlo La Spina, Alessandro Rabiolo, Giuseppe Querques, Francesco Bandello

**Affiliations:** 1 Ophthalmology Unit, Ospedale di Desio, ASST Monza, Desio, Italy; 2 Ophthalmology Unit A, Turin Eye Hospital, ASL “Città di Torino”, Turin, Italy; 3 Department of Ophthalmology, IRCCS San Raffaele Scientific Institute, University Vita Salute San Raffaele, Via Olgettina 60, 20132 Milan, Italy

**Keywords:** Heads-up surgery, Retinal detachment, Vitrectomy

## Abstract

Here we present procedural and safety results from our initial experience using 3D heads-up vitrectomy for retinal detachment surgery. The comparison with our outcomes from previous standard surgery shows how the new 3D system seems safe and effective.

**Electronic supplementary material:**

The online version of this article (doi:10.1186/s40942-017-0099-2) contains supplementary material, which is available to authorized users.

## Introduction

The application of a three-dimensional imaging system (3D) to retinal surgery is the latest and more exciting advance in the field. Previous studies demonstrated the technical feasibility of 3D surgery [1, 2]. This new imaging system offers many advantages including a more physiological “heads-up” position for the surgeon, a very high image definition even at wider magnifications, ad hoc digital filters (e.g., to enhance vitreous visibility), lower endoillumination levels [3], and the same view between the audience (e.g., nurses, fellows, students) and the surgeon [1, 2]. Following their release in the market, many surgeons started to operate routinely using a 3D system. Although the 3D system is different from traditional surgery, no direct comparison between these two methodologies is provided by the current literature.

The present study aimed to compare the outcomes of a 3D system with traditional ones in the setting of retinal detachment (RD) surgery.

## Methods

**Fig. 1:**
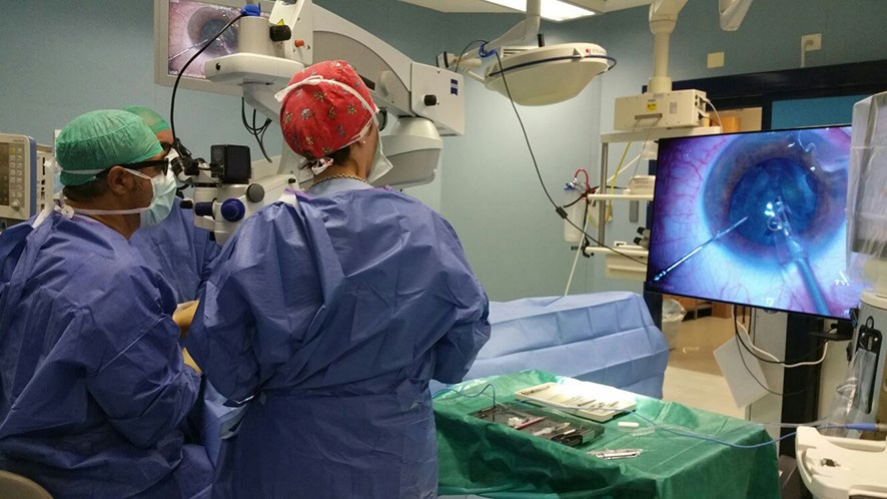
Surgeon and his assistants during the surgery using a heads-up 3D vision system

From April 1st, 2017, all RD cases were routinely performed using the Ingenuity 3D system (Alcon, Fort Worth, Texas, USA). All cases were performed using a standard technique (25 gauge vitrectomy plus endolaser and gas/silicone oil tamponade) by a single experienced vitreoretinal surgeon (MC). An additional movie shows a case of rhegmatogenous RD operated using the 3D system (see Additional file 1). The surgical technique did not differ from previous surgery performed using a traditional system. We compared the 3D Group with a control group including all cases performed from January the 1st 2017 to March the 31st 2017 (Fig. 1).

## Results

We collected a total of 7 cases in the 3D group. Six (86%) of them were primary simple cases, while 1 (14%) of them was a complex recurrent one. Traditional group consisted of 15 cases of which 1 (7%) was a complicated recurrent case, and 14 (93%) were simple primary cases.

Immediate procedural success (defined as complete re-attachment at day 1 after surgery) was achieved in all cases for both groups. At 30 days after surgery, no cases of re-detachment were recorded in the 3D group, while one case occurred in the control group (p = 0.74). None of the eyes experienced any major post-operative complication. Five patients in the 3D group and two patients in the control one required medications for augmented intraocular pressure at day 1 (p = 0.56). The mean ± standard deviation of surgery time was 55 ± 35 min for the 3D group and 62 ± 28 min for the control group (p = 0.07). Mean endoillumination power during the procedure was 10 and 45% for groups 3D and control, respectively (p < 0.0001). In 9 eyes (60%) of the control group, diluted Triamcinolone was injected to improve the visualization of vitreous remnants. Conversely, vitreous staining was not performed in any eye of the 3D group (p = 0.01).

## Discussion

RD surgery is one of the most challenging procedures for the vitreo-retinal surgeon. Proper visualization during the surgery may influence the outcome dramatically. Some years ago, the introduction of non-contact wide field viewing systems led to considerable improvement in the field [4]. The 3D systems provide a new viewing experience and offer several advantages compared to traditional systems (Table 1). This is, to the best of our knowledge, the first report to investigate the outcomes of RD repair using a 3D system. In our small initial series, we found no significant differences in procedural success rates compared to traditional surgery. Interestingly, the 3D system allowed avoiding triamcinolone vitreous staining. This could be related to the magnified high-resolution image, and to the use of digital filters (e.g., green filter) that enable the surgeon to visualize the vitreous remnants optimally. The avoidance of some surgical steps (i.e., vitreous staining) may explain the non-significant tendency towards shorter procedure time. However, we acknowledge that surgical time is influenced by the difficulty of each singular case. The homogeneity cannot be guaranteed by a non-randomized retrospective comparison and, therefore, differences in operating time between the two systems should be interpreted with caution.

**Table 1:** Advantages of 3D system for retinal detachment surgery

Illumination	Digital enhancement of the image, lower illumination levels and, thus, reduced risk of phototoxicity
Depth of field	Enhance depth of field and amplified stereopsis allows better wider view since multiple planes are contemporary in focus
HDRi	Reduced glare of the instruments and even image brightness
Digital filtering	Filters enable the surgeon to have a better view of intraocular structures (e.g. green filter for vitreous or blood)
Education	Surgical team has the same view of the surgeon
ergonomics	Surgeon maintains an heads up position with a less rigid and more comfortable posture
Less asthenopia	Viewing through the eyepiece for a long time may induce asthenopia due to concentration and prolonged accommodation. Since 3D systems does not require near vision, it may reduce asthenopia

*3D* three-dimensional; *HDRI* high dynamic range imaging

The digital image enhancement enabled to reduce the endoillumination power significantly minimizing the phototoxicity to the retinal pigmented epithelium cells [5]. All cases were performed using a light probe and, therefore, 3D visibility using other systems (i.e., chandelier light pipe, diaphanoscopic extraocular illumination) remains still indeterminate.

Due to the retrospective design, some variables (e.g., visualization of retinal holes and epiretinal membranes, visibility under air) were not recorded in our clinical charts and, therefore, we are not able to provide a direct comparison between the two imaging modalities for these points. We feel that 3D system, however, is not inferior to conventional surgery under these specific aspects. Due to the enhanced depth of field, multiple planes are contemporary in focus and this allows to have a wide field of view. Although the system has a latency 80 ms later than standard microscope, no significant delay was noted when performing intraocular procedures.

This report reflects the initial experience of a single center. Our findings need to be confirmed in larger multicenter, prospective studies. Nonetheless, in our experience, 3D visualization system seems to be as safe and effective as the traditional one providing all the advantages of digital over an analogic platform. In addition, it permits to avoid phototoxic risks related to endoillumination.

## Additional file

**Additional file 1.** 25-G rhegmatogenous retinal detachment. The additional movie file shows a 25-G pars plana vitrectomy, plus endolaser and gas tamponade in a pseudophakic patient for rhegmatogenous retinal detachment using the 3D visualization system.

## Abbreviation

RD: retinal detachment
